# Simulation of Genome-Wide Evolution under Heterogeneous Substitution Models and Complex Multispecies Coalescent Histories

**DOI:** 10.1093/molbev/msu078

**Published:** 2014-03-19

**Authors:** Miguel Arenas, David Posada

**Affiliations:** ^1^Centre for Molecular Biology “Severo Ochoa,” Consejo Superior de Investigaciones Científicas (CSIC), Madrid, Spain; ^2^Department of Biochemistry, Genetics and Immunology, University of Vigo, Vigo, Spain

**Keywords:** heterogeneous substitution models, multispecies coalescent, molecular adaptation, molecular evolution

## Abstract

Genomic evolution can be highly heterogeneous. Here, we introduce a new framework to simulate genome-wide sequence evolution under a variety of substitution models that may change along the genome and the phylogeny, following complex multispecies coalescent histories that can include recombination, demographics, longitudinal sampling, population subdivision/species history, and migration. A key aspect of our simulation strategy is that the heterogeneity of the whole evolutionary process can be parameterized according to statistical prior distributions specified by the user. We used this framework to carry out a study of the impact of variable codon frequencies across genomic regions on the estimation of the genome-wide nonsynonymous/synonymous ratio. We found that both variable codon frequencies across genes and rate variation among sites and regions can lead to severe underestimation of the global *dN*/*dS* values. The program *SGWE*—Simulation of Genome-Wide Evolution—is freely available from http://code.google.com/p/sgwe-project/, including extensive documentation and detailed examples.

Computer simulations are important for different purposes in molecular evolution. For example, they can be used for hypothesis testing, to evaluate and validate analytical methods, or to estimate evolutionary parameters (see Arenas 2012; Hoban et al. 2012). To our knowledge, only a few simulators of genome-wide evolution have been developed so far (table 1). Tools like *EvolSimulator* (Beiko and Charlebois 2007) and *ALF* (Dalquen et al. 2012) are able to actually simulate genomic events like duplication or rearrangement, while others basically simulate multiple genomic regions. Except *ALF*, current genome-wide simulators assume a constant substitution process across the entire genome, which might seem overly simplistic (Arbiza et al. 2011). In the case of *ALF* however, the user has to specify by hand the substitution model for any predefined genomic region, which can be too tedious for more than a few genes. Furthermore, in all these simulators, specific parameter values need to be specified by the user, which is not always an easy task.

**Table 1.** Genome-Wide Simulation Software.

Program	Class	Evolutionary Process	Substitution Process	Variable *dN/dS* across Sites and Branches	Rate Variation	Indels	Homogeneous/ Heterogeneous Substitution Model across Regions	Reference
SIMCOAL2 and Fastsimcoal	Coalescent	D, M, R	N: JC, K2P	No	No	No	Homogeneous	Excoffier et al. (2000); Excoffier and Foll (2011)
GenomePop	Forward	D, M, R, S	N: GTR; Cod: MG94	No	No	No	Homogeneous	Carvajal-Rodriguez (2008)
EvolSimulator	Birth–death process	D, M, L, S	N: GTR; C: Nt^a^; A: user defined	No	G_sites_^b^	No	Homogeneous	Beiko and Charlebois (2007)
GSIMULATOR package	Phylogenetic	—	N: GTR; C: EM; A: Secondary structure	No	No	Yes	Homogeneous	Varadarajan et al. (2008)
ALF	Birth–death process and phylogenetic	M, L	N: GTR; C: GY94 (M0,M2,M3,M8) and EM; A: 5 EM^c^	Yes	G_sites_,^b^ I	Yes	Homogeneous/heterogeneous^d^	Dalquen et al. (2012)
SGWE	Coalescent and phylogenetic	D, N, R	N: GTR; C: GY94 (M0-M13), MG94, HB and EM; A: 16 EM^e^	Yes	G_sites_^b^ and/or G_regions_, I	Yes	Heterogeneous	This study

Note.—The column “Class” includes phylogenetic (where a phylogeny is user-specified), forward, birth–death, and coalescent approaches. The column “Evolutionary process” describes the implemented evolutionary scenarios: D (demographics), M (population structure and migration), R (recombination), L (lateral gene transfer), and S (selection). The column “Substitution process” refers to N (nucleotide), C (codon), and A (amino acid) substitution/replacement models. EM means “empirical model,” and it is indicated whether the model is fixed along the genome (homogeneous) or can change among genomic regions (heterogeneous). The column “Rate variation” indicates whether different sites can evolve under different rates (G: gamma distribution; I: proportion of invariable sites) and whether this level of heterogeneity can change across site positions (G_sites_) and/or genomic regions (G_regions_). The column “Indels” indicates the consideration of insertion and deletion events.

^a^Coding sequences are simulated through nucleotide substitution models just avoiding stop codons.

^b^The rate of variation among sites can be user-specified.

^c^Amino acid models implemented in *ALF*: JTT, GCB, LG, WAG, CustomP.

^d^A maximum of three genomic regions based on different substitution models can be simulated.

^e^Amino acid models implemented in *SGWE*: Blosum62, CpRev, Dayhoff, DayhoffDCMUT, HIVb, HIVw, JTT, JonesDCMUT, LG, Mtart, Mtmam, Mtrev24, RtRev, VT, WAG, user-specified. See references in the supplementary material, Supplementary Material online.

Here, we present a simulation framework called *SGWE* (Simulation of Genome-Wide Evolution) that is able to simulate multigene data sets accounting for heterogeneous evolution across genomic regions. Importantly, this heterogeneity is controlled by the user through the specification of prior statistical distributions from which specific parameter values are sampled for each genomic region and replicate. Furthermore, evolutionary histories can be specified by the user or simulated by the multispecies coalescent with recombination—including hotspots and coldspots—demographics, and migration, among other evolutionary scenarios. We used this simulation framework to study the impact of variable codon frequencies across regions on the estimation of the *dN*/*dS* ratio.

## New Approaches: SGWE

**Fig. 1.**
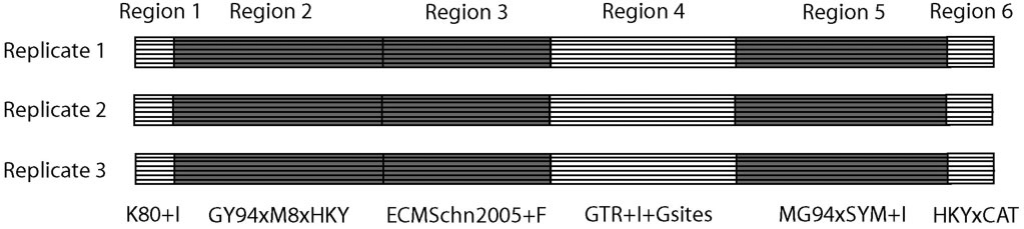
Depiction of three genome alignments simulated with *SGWE*. Each genome alignment contains six regions, printed with white and gray background to describe noncoding and coding regions, respectively. “+I” indicates proportion of invariable sites, and “+Gsites” indicates heterogeneity across sites according to a gamma distribution. “ECMSchn2005” indicates the empirical codon model by Schneider et al. (2005). “+F” indicates empirical frequencies (e.g., user-specified) are considered. “CAT” indicates that frequencies change across sites within a region.

*SGWE* simulates genome-wide sequence evolution through the specification of genome-wide parameters and prior distributions for local parameters governing the evolution of the different genomic regions. Supplementary table S1, Supplementary Material online, shows a list of the different evolutionary scenarios that can be implemented in *SGWE*. The simulation procedure consists of two steps. In the first step, the user can specify every aspect of the simulation through a user-friendly Graphical User Interface (GUI), with the possibility of loading up to ten prespecified scenarios. The GUI window includes a series of frames where the user can define target evolutionary scenarios and the underlining prior distributions for the different parameters. In the second step, *SGWE* simulates each genomic region according to the specific genome-wide and local parameters sampled from the prior distributions. Each simulated replicate consists of a set of aligned genomes (fig. 1). Internally, each genomic region is simulated under the multispecies coalescent with recombination, including intracodon and hotspot recombination (Wiuf and Posada 2003; Arenas and Posada 2007; Arenas and Posada 2010), demographic periods, exponential growth, and several migration models with constant or time-dependent migration rates (Wright 1931; Kimura and Weiss 1964; Hudson 1998), longitudinal sampling (i.e., noncontemporaneous sequences) (Drummond et al. 2002), under multiple nucleotide, codon, and protein substitution/replacement models. SGWE implements nucleotide substitution models like GTR (Tavaré 1986) plus invariable sites and gamma-distributed rate variation among sites (i.e., the GTR+I+G model) (Yang 1994) and special cases of it. In addition, the user can select codon models like GY94 × M0-M10 (Yang et al. 2000; Anisimova et al. 2001) where *dN/dS* can vary across branches, MG94 (Muse and Gaut 1994), Halpern and Bruno (HB) (Halpern and Bruno 1998; Holder et al. 2008), or different empirical codon models (Schneider et al. 2005; Kosiol et al. 2007). Finally, SGWE also implements 16 empirical matrices and the CAT model (Lartillot and Philippe 2004) for amino acid replacement with variable frequencies across sites.

Although *SGWE* implements a large variety of evolutionary scenarios and substitution models, it does not directly implement indel evolution. This is mainly based on the complexity of simulating the coalescent with recombination (Hudson and Kaplan 1988) with indels, because the former requires a fixed sequence length. However, in a way that is transparent to the user, *SGWE* is able to call *INDELible* (Fletcher and Yang 2009), a simulation software that implements a wide set of models of indel evolution along a fixed phylogeny.

The *SGWE* pipeline is written in Java, C, Perl, and R and is freely available from http://code.google.com/p/sgwe-project/ (last accessed March 4, 2014). The downloadable package includes executable files, source code, documentation, and a variety of practical examples. Furthermore, SGWE’s coalescent simulator can be used on its own on the command line for single locus simulations. This simulator is written in C, can run in parallel, and is freely available from http://code.google.com/p/coalevol/ (last accessed March 4, 2014).

## Benchmarking

The implementation of SGWE was validated using theoretical expectations and/or comparisons with other simulation/analytical software. For example:

- Different simulation outcomes, like the time to the most recent common ancestor (TMRCA) or the number of recombination events, were in agreement with theoretical expectations and with those obtained under the same settings using *ms* (Hudson 2002).
- Simulated genealogies under diverse evolutionary scenarios were accurately reconstructed using *Phyml* (Guindon and Gascuel 2003).
- Generating nucleotide and amino acid substitution models were correctly identified using *jModelTest* (Posada 2008) and *ProtTest* (Abascal et al. 2005), respectively.
- Simulated *dN/dS* values were accurately estimated with *PAML*, *Hyphy* (Kosakovsky Pond et al. 2005), and *SNAP* (Korber 2000).

Further details are given in supplementary note S1, Supplementary Material online.

## An Example: Influence of Heterogeneous Codon Frequencies and Substitution Rates on *dN*/*dS* Estimates

To illustrate a potential use of *SGWE*, we studied the influence of variable transition/transversion rates ratio (*ti*/*tv*) and variable codon frequencies on the estimation of *dN*/*dS* (e.g., Oleksyk et al. 2010; Kjeldsen et al. 2012; Smith et al. 2013). Using SWGE, we simulated genome alignments were *dN*/*dS* was kept constant across the different genomic regions, but *ti*/*tv* and the codon frequencies varied among them. Then, we estimated *dN*/*dS* assuming that all parameters were constant along the different genomic regions.

**Fig. 2.**
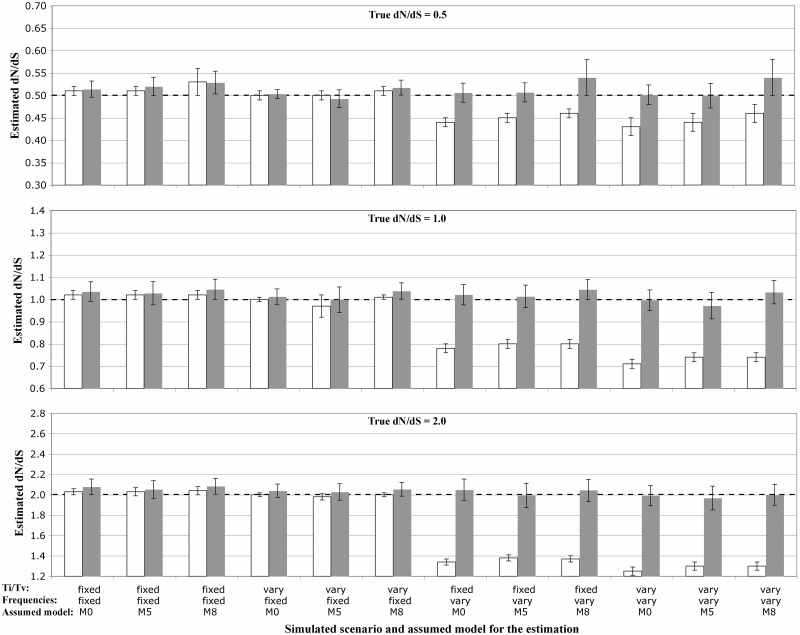
Influence of variable codon frequencies and variable *ti*/*tv* across regions on the estimation of the genome-wide *dN*/*dS* when the true *dN*/*dS* value is 0.5, 1.0, and 2.0. The horizontal dashed black line indicates the simulated *dN*/*dS* value. White bars indicate the estimated *dN*/*dS* from the entire genome, while the gray bars display the averaged *dN*/*dS* across regions. Error bars indicate 95% confidence intervals.

**Fig. 3.**
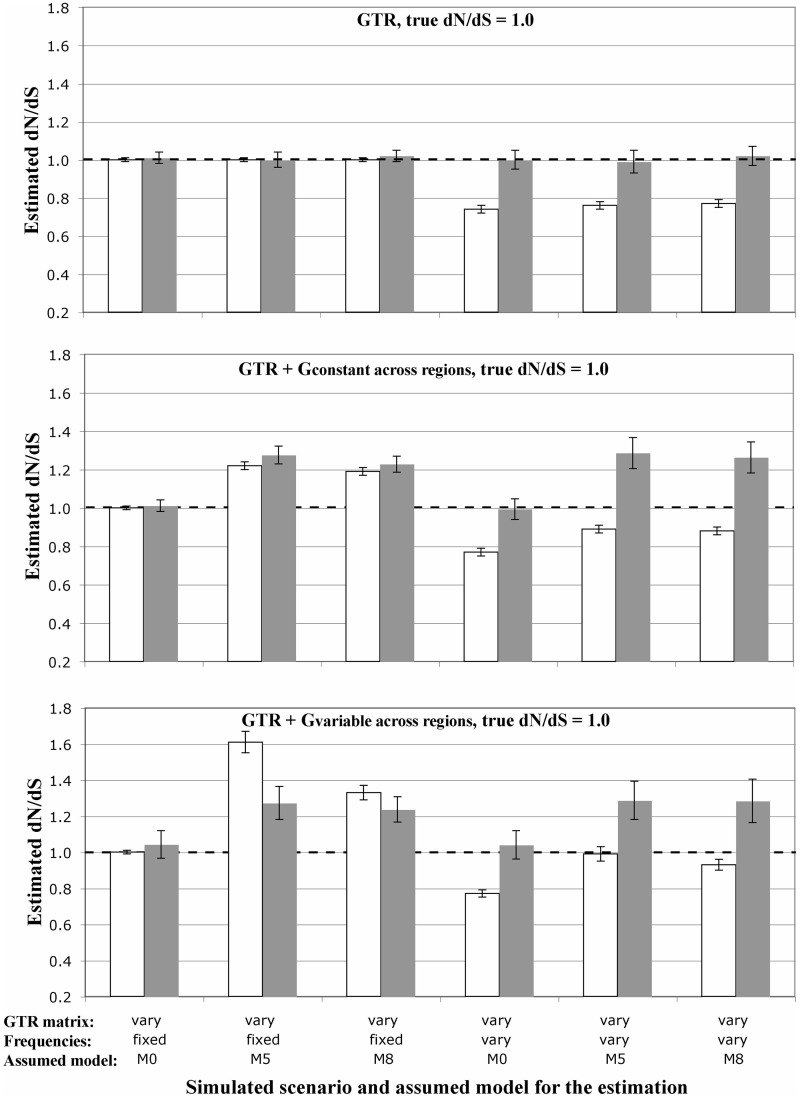
Influence of variable codon frequencies, variable transition rates, and gamma-distributed rate variation among sites and across regions on the estimation of the genome-wide *dN*/*dS* when the true *dN*/*dS* value is 1.0. The horizontal dashed black line indicates the true, simulated value. White bars indicate the estimated *dN*/*dS* from the entire genome, while the gray bars display the averaged *dN*/*dS* across regions. Error bars indicate 95% confidence intervals.

In the absence of rate variation among sites or regions, when only the *ti*/*tv* (fig. 2) or the GTR matrices (fig. 3 and supplementary figs. S1 and S2, Supplementary Material online, upper plots) varied across regions, the *dN*/*dS* estimates were very accurate. On the contrary, when the codon frequencies varied across regions, the global *dN*/*dS* was consistently underestimated (figs. 2 and 3 and supplementary figs. S1 and S2, Supplementary Material online; white bars). For example for simulated values of 2.0, 1.0, and 0.5, the average *dN*/*dS* estimates were 1.25 ± 0.04, 0.71 ± 0.02, and 0.43 ± 0.01, respectively (fig. 2). However, if the average of the local *dN*/*dS* estimates for each region was considered as an estimate of global *dN*/*dS*, the bias was not observed (figs. 2 and 3 and supplementary figs. S1 and S2, Supplementary Material online, upper plots; gray bars).

Introducing rate variation among sites and regions resulted in a very complex picture, where different combination of parameters resulted in underestimates or overestimates of the simulated *dN/dS* value (fig. 3 and supplementary figs. S1 and S2, Supplementary Material online). When codon frequencies were constant across regions, the M0 model resulted in accurate *dN/dS* estimates, but estimates under models M8 and especially M5 were biased upward. When codon frequencies varied across regions, the *dN/dS* estimates were biased downward. In general, these biases were more pronounced when different regions had distinct levels of among-site rate variation (fig. 3 and supplementary figs. S1 and S2, Supplementary Material online, lower plots).

## Discussion

Sequence evolution across different genomic regions can be highly heterogeneous (e.g., Gibbs et al. 2007; Arbiza et al. 2011). Simulation and empirical studies tend to ignore this heterogeneity and assume that multigene data sets evolve under one or very few substitution models. *SGWE* implements a simulation framework to simulate genome-wide sequence evolution that accounts for evolutionary heterogeneity in time and (sequence) space, better reflecting the evolutionary process shaping real data. A key aspect of *SGWE* is that the heterogeneity of the whole evolutionary process can be parameterized according to statistical prior distributions specified by the user, allowing much needed flexibility. We believe that *SGWE* is complementary to other comprehensive tools like *ALF*, which implement a range of genomic events not included in *SGWE* but which cannot handle easily variation across regions and does not currently simulate population-genetic events such as recombination or lineage sorting within species trees.

At this point, SGWE’s coalescent simulator and *INDELible* cannot run at the same time in a given simulation experiment, so recombination simulations cannot be run with indels, for example. Which one to choose depends on the particular biological scenario that the user wants to implement. In general, coalescent simulations should be more useful in intraspecific scenarios or in interspecific situations with incomplete lineage sorting, and phylogenetic simulations with *INDELible* should be more appropriate for interspecific evolution with no phylogenomic incongruence (i.e., where gene trees across the genome are equal). A detailed list of the capabilities implemented in *SGWE* is shown in the supplementary table S1, Supplementary Material online.

As an example of the use of *SGWE*, we studied the impact of variable codon frequencies and among-site rate variation across genomic regions on the estimation of *dN*/*dS*. It is well known that protein-coding sequences usually show variable frequencies across protein regions as a consequence of protein folding, solvent accessibility, and protein function (e.g., Goldman et al. 1998; Lio and Goldman 1999; Liberles et al. 2012; Arenas et al. 2013). While different models of sequence evolution exist capable of accounting for this heterogeneity (Bruno 1996; Halpern and Bruno 1998; Pagel and Meade 2004; Holder et al. 2008), these are seldom used in real data—at least at the DNA and codon level—probably because they are computationally very intensive. Our simulation experiments with *SGWE* show that, in general, variable codon frequencies can result in the underestimation of genome-wide *dN*/*dS* values, while rate variation among sites and regions seem to have the opposite effect. On the other hand, the average of the regional estimates seems to be a good approximation of the genome-wide *dN*/*dS* value. The fact that model misspecification cause error in *dN*/*dS* estimation is hardly surprising, but our simulations confirm this expectation and more importantly quantify the bias. Indeed, most studies do not rely on a single, genome-wide *dN*/*dS* estimate, but they might still try to obtain single estimates from single genomic fragments that in fact could include distinct substitution models (i.e., fragments that encompass multiple genomic regions) and therefore be the subject of similar biases.

Here, we are under a model underfitting scenario, where the model assumed for parameter estimation is always simpler than the true model used to simulate the data. It is known that this circumstance usually leads to parameter underestimation, for example of the branch lengths or of the *ti*/*tv* ratio (Tamura 1992; Lemmon and Moriarty 2004). In our simulations, the reasons why some model violations can induce underestimation of the global *dN*/*dS* and others overestimation are not straightforward. Moreover, different misspecifications of the assumed model operate here in opposite directions. Ignoring variable codon frequencies seems to push the *dN*/*dS* estimates downward. In particular, we could see that this was due to the simultaneous underestimation of *dN* and the overestimation of *dS*. Also, increasing the number of variable regions accentuated this bias (data not shown). Codon frequency biases have been shown before to induce underestimation of *dN/dS* for some ML methods (Yang and Nielsen 2000). Accordingly, variation in GC content along a sequence seems to reduce the number of true positives of the branch-site test (Gharib and Robinson-Rechavi 2013).

On the other hand, ignoring rate variation among sites, especially when this change among regions, biased the *dN*/*dS* estimates upward under the M5 and M8 models, but not under the M0 model. The M5 model assumes a gamma distribution for *dN*/*dS* variation among sites, while M8 adds to M5 a proportion of sites with *dN*/*dS* > 1. In the simulations, *dN*/*dS* was always constant across regions, but the bias appeared when the substitution rate changed within regions, and specially when it did it in different way in different regions (i.e., according to different gamma distributions). The exact reasons for this are not straightforward, although it is known that the M5 and M8 models can be less conservative than the M0 model (Yang et al. 2000; Metzger and Thomas 2010).

Apart from simulation studies like the one implemented here, *SGWE* could also be used to benchmark species tree estimation, to understand the interactions between different evolutionary forces at the genome-wide level or to estimate evolutionary parameters and perform model choice using approximate Bayesian computation (Beaumont 2010; Lopes et al. 2014).

## Material and Methods

### Simulation of Variable Codon Frequencies, *ti*/*tv*, and Substitution Rates across Genomic Regions

Gene genealogies for each genomic region were simulated under the coalescent assuming a constant effective population size of 1,000 and a sample size of 15 individuals. Each individual genome was composed of 15 genomic regions or genes, with 150 codons each. Genomic sequences were evolved over these genealogies assuming a GY94 × M0 codon model under three genome-wide *dN*/*dS* values: 0.5, 1, and 2. Transition/transversion (*ti*/*tv*) ratios were either fixed to 0.5 or varied across regions according to a Uniform distribution truncated between 0.5 and 15. Substitution rates (A-C, A-G, A-T, C-G, C-T, G-T) varied across regions according to a Dirichlet distribution D(6,16,2,8,20,4) that was then scaled with the last rate. Scenarios with rate variation across sites were simulated according to a gamma distribution (+G) with shape 0.7. Scenarios where this gamma distribution varied across regions drew the different gamma shapes from to an exponential distribution with mean 2.0 and truncated between 0.5 and 5.0. Such parameter values are typical of RNA virus like HIV-1 (Carvajal-Rodriguez et al. 2006). Codon frequencies were specified according to the nucleotide frequencies at each codon position. The latter were either constant (0.25 for each codon position) or varied across regions according to a Dirichlet distribution (D(1,1,1,1) for each codon position). For each scenario, we simulated a total of 100 genome alignments.

### Estimation of *dN*/*dS*

Genome-wide *dN/dS* values were estimated using the *codeml* program from PAML (Yang 2007) under the GY94 × M0 (constant *dN*/*dS*), GY94 × M5 (*dN*/*dS* follows a Gamma distribution), and GY94 × M8 (two categories, Beta distribution + *dN*/*dS* ≥ 1) codon models (Yang et al. 2000). We choose *PAML* because it is a well-known, commonly used, comprehensive, and validated software to estimate *dN/dS*. The assumed codon frequencies were calculated as a function of the empirical nucleotide frequencies at each codon position. As a sanity check, similar *dN/dS* estimates under GY94 × M0 codon model were obtained when we used *Hyphy* instead of *PAML* (supplementary fig. S4, Supplementary Material online).

## Supplementary Material

Supplementary figures S1–S3 and tables S1–S6 are available at *Molecular Biology and Evolution* online (http://www.mbe.oxfordjournals.org/).

Supplementary Data
