# Supplementary material for: Simulation of Genome-Wide Evolution under Heterogeneous Substitution Models and Complex Multispecies Coalescent Histories
Source: Mol Biol Evol. 2014 Mar 19;31(5):1295–301. doi: 10.1093/molbev/msu078 (PMC3995339; doi:10.1093/molbev/msu078)
Supplement: Supplementary Data [file supp_31_5_1295__index.html]

Simulation of Genome-wide Evolution under Heterogeneous Substitution models and Complex Multispecies Coalescent Histories — Simulation of Genome-Wide Evolution under Heterogeneous Substitution Models and Complex Multispecies Coalescent Histories — Simulation of Genome-Wide Evolution under Heterogeneous Substitution Models and Complex Multispecies Coalescent Histories — Supplementary Data 

# Simulation of Genome-Wide Evolution under Heterogeneous Substitution Models and Complex Multispecies Coalescent Histories

## Supplementary Data

files

**Files in this Data Supplement:**

- Supplementary Data - doc file
