# Supplementary material for: Simulation of Genome-Wide Evolution under Heterogeneous Substitution Models and Complex Multispecies Coalescent Histories
Source: Mol Biol Evol. 2014 Mar 19;31(5):1295–301. doi: 10.1093/molbev/msu078 (PMC3995339; doi:10.1093/molbev/msu078)
Supplement: Supplementary Data [file supp_msu078_SGWE_MBE_supmat_REV2_2.doc]

**S****upplementary Material**

***Simulation of Genome-wide Evolution under Heterogeneous Substitution models and Complex Multispecies Coalescent Histories***

Miguel Arenas*^*^* and David Posada^#^

*^*^ Centre for Molecular Biology “Severo Ochoa”, Consejo Superior de Investigaciones Científicas (CSIC), Madrid, Spain.*

*^#^ Department of Biochemistry, Genetics and Immunology, University of Vigo, Vigo, Spain.*

**Email addresses:**

MA: [marenas@cbm.uam.es](mailto:marenas@cbm.uam.es)

DP: [dposada@uvigo.es](mailto:dposada@uvigo.es)


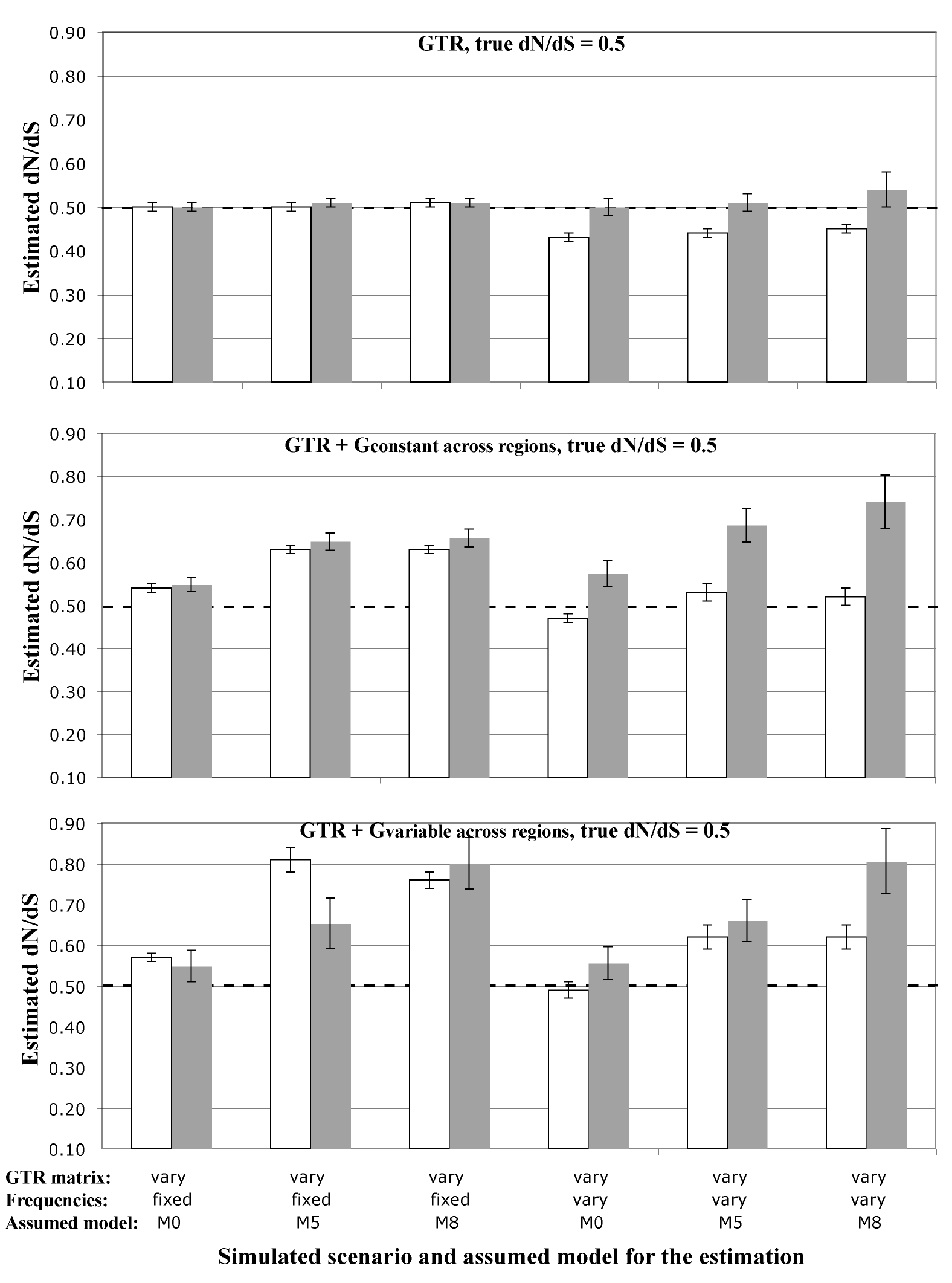


**Figure S1**. Influence of variable codon frequencies, variable transition rates and, gamma-distributed rate variation among sites and across regions on the estimation of the genome-wide *dN*/*dS* when the true *dN*/*dS* value is 0.5. The horizontal dashed black line indicates the true, simulated value. White bars indicate the estimated *dN*/*dS* from the entire genome, while the grey bars display the averaged *dN*/*dS* across regions. Error bars indicate 95% confidence intervals.


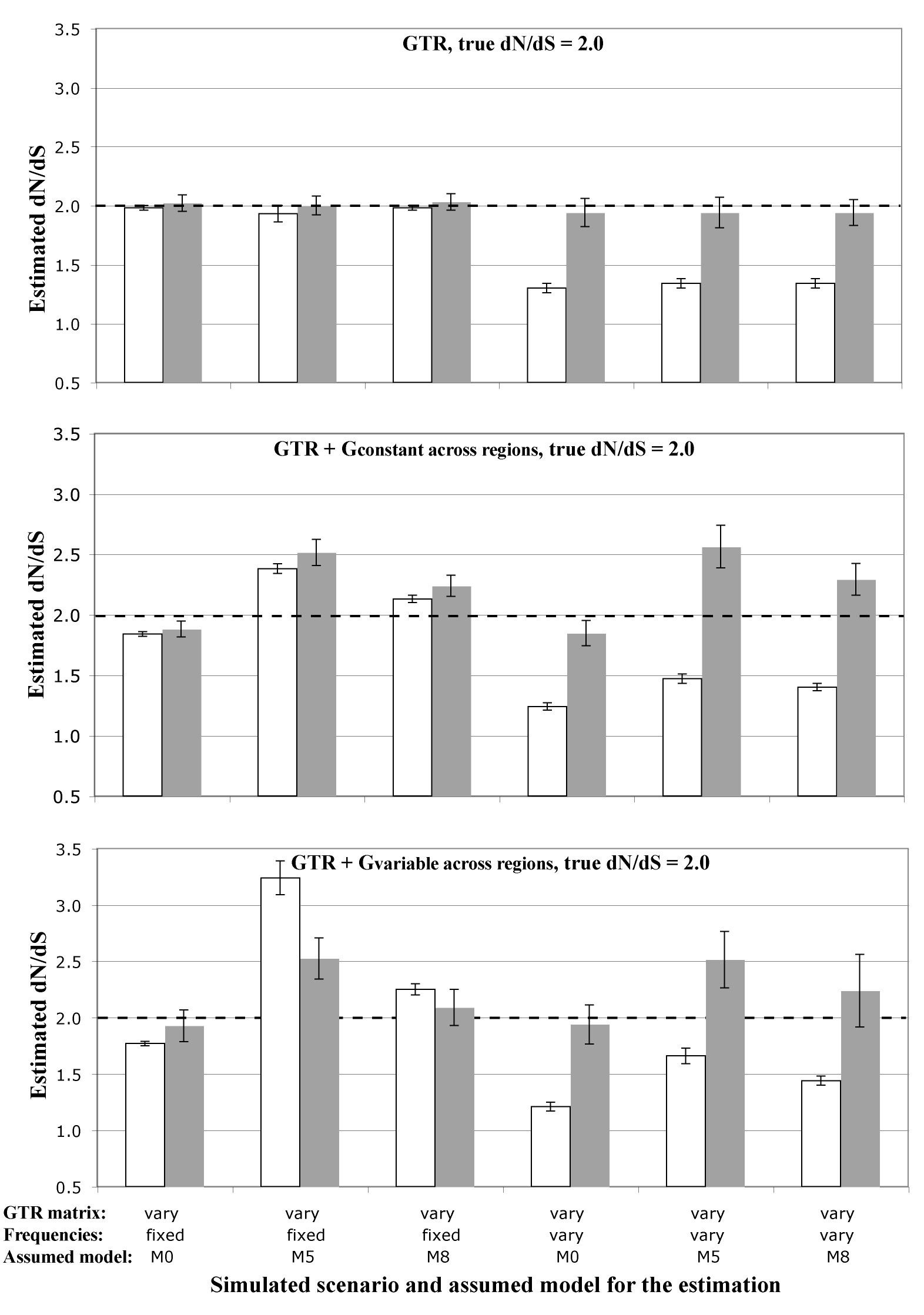


**Figure S2.** Influence of variable codon frequencies, variable transition rates and, gamma-distributed rate variation among sites and across regions on the estimation of the genome-wide *dN*/*dS* when the true *dN*/*dS* value is 2.0. The horizontal dashed black line indicates the true, simulated value. White bars indicate the estimated *dN*/*dS* from the entire genome, while the grey bars display the averaged *dN*/*dS* across regions. Error bars indicate 95% confidence intervals.


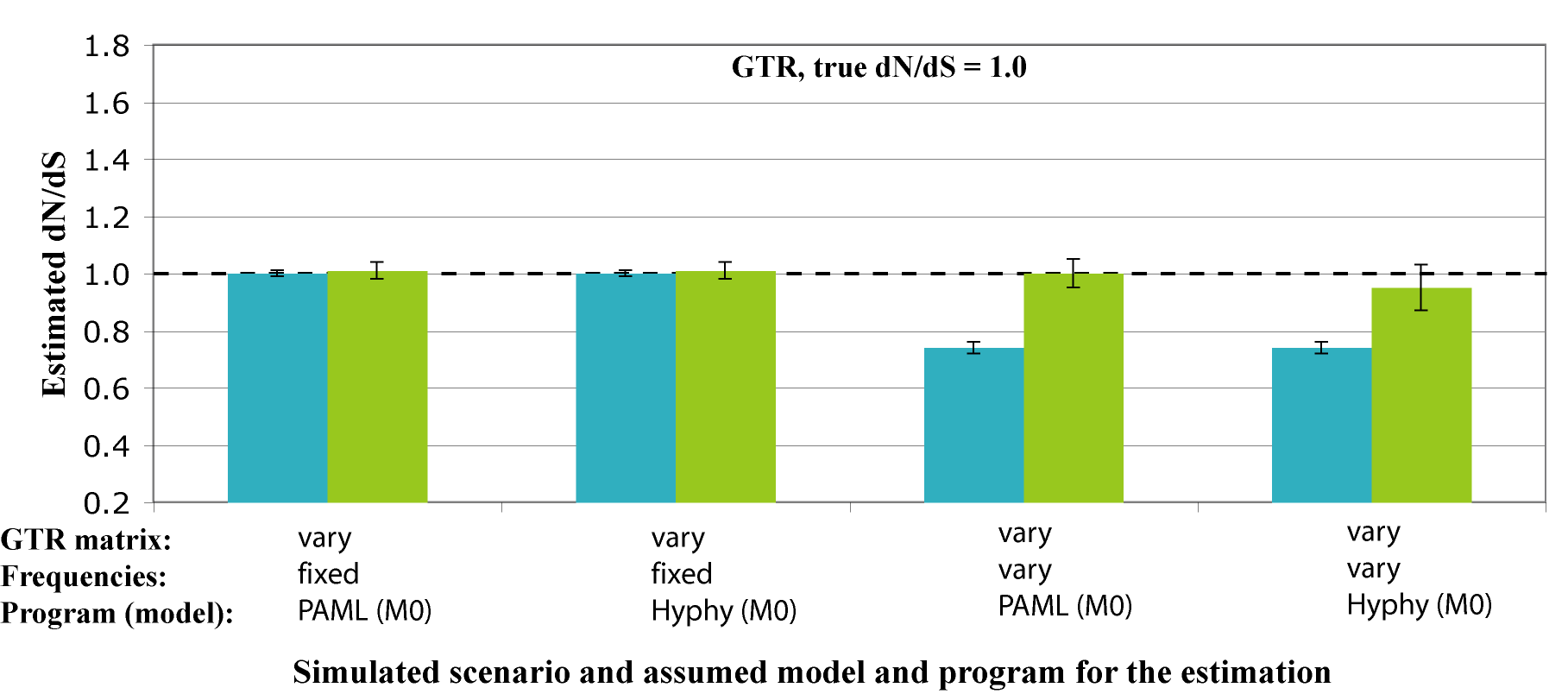


**Figure S3.** Influence of variable codon frequencies, variable transition rates and, gamma-distributed rate variation among sites and across regions on the estimation of the genome-wide *dN*/*dS* when the true *dN*/*dS* value is 1.0 using *PAML* and *Hyphy*. The horizontal dashed black line indicates the true, simulated value. Blue bars indicate the estimated *dN*/*dS* from the entire genome, while the green bars display the averaged *dN*/*dS* across regions. Error bars indicate 95% confidence intervals.

**Table S1.** A full list of capabilities implemented in *SGWE*. Notice that some of the conditions cannot be applied simultaneously because SGWE uses two sequence simulators (its own, *CoalEvol*, and *INDELible* (Fletcher and Yang 2009)).

| **Evolutionary feature** | **Capability** |
| --- | --- |
| Coalescent history *(CoalEvol)* | Haploid / Diploid data |
|  | Demographics (growth rate and demography by using temporal periods) |
|  | Multispecies history / population structure |
|  | Migration (island (Hudson 1998), stepping-stone (Kimura and Weiss 1964) and continent-island (Wright 1931) models) |
|  | Homogeneous Recombination (constant within a genomic region) |
|  | Homogeneous Recombination (variable across genomic regions) |
|  | Heterogeneous recombination (hotspots and coldspots), following (Wiuf and Posada 2003) |
|  | Intracodon recombination (only for codon models) following (Arenas and Posada 2010) |
|  | Substitution rate (constant within a genomic region) |
|  | Substitution rate (variable across genomic regions) |
|  | Longitudinal sampling, (see, Navascues, Depaulis, and Emerson 2010) |
|  | Outgroup |
| User-specified input tree | A tree or a set of trees for a genomic region *(CoalEvol)* |
|  | A tree or a set of trees for the genome *(CoalEvol and INDELible)* |
| Non-coding DNA substitution | All nucleotide substitution models (JC (Jukes and Cantor 1969), …, GTR (Tavaré 1986)), model and parameters constant within a genomic region *(CoalEvol and INDELible)* |
|  | All nucleotide substitution models (JC, …, GTR), model and parameters variable across genomic regions *(CoalEvol and INDELible)* |
|  | Heterogeneous rate across sites (+Gsites) *(CoalEvol and INDELible)* |
|  | Heterogeneous rate across genomic regions (+Gregions) *(CoalEvol)* |
|  | Heterogeneous rate across both sites and regions (+Gregions +variable Gsites) *(CoalEvol)* |
|  | Proportion of invariable sites within a genomic region (+I) *(CoalEvol and INDELible)* |
|  | Proportion of invariable sites variable across genomic regions (variable +I) *(CoalEvol and INDELible)* |
|  | Nucleotide frequencies constant within a genomic region *(CoalEvol and INDELible)* |
|  | Nucleotide frequencies variable across genomic regions *(CoalEvol and INDELible)* |
|  | Variable nucleotide frequencies across sites by categories (CAT) (Lartillot and Philippe 2004), within a genomic region *(CoalEvol)* |
|  | Variable nucleotide frequencies across sites by categories (CAT), and variable across genomic regions *(CoalEvol)* |
|  | Transition transversion ratio rate constant within a genomic region *(CoalEvol and INDELible)* |
|  | Transition transversion ratio rate variable across genomic regions *(CoalEvol and INDELible)* |
|  | Rates of change constant within a genomic region *(CoalEvol and INDELible)* |
|  | Rates of change variable across genomic regions *(CoalEvol and INDELible)* |
|  | Nucleotide indels (for insertion and deletion events: rates and models (NB, LAV, POW continuous, POW discrete and user-specified) (Fletcher and Yang 2009)), model and parameters constant within a genomic region *(INDELible)* |
|  | Nucleotide indels (for insertion and deletion events: rates and models (NB, LAV, POW continuous, POW discrete and user-specified)), model and parameters variable across genomic regions *(INDELible)* |
| Coding DNA substitution | GY94 codon models (Goldman and Yang 1994) with heterogeneous dN/dS across sites (Yang et al. 2000; Anisimova, Bielawski, and Yang 2001), model and parameters constant within a genomic region *(CoalEvol and INDELible)* |
|  | GY94 codon models with heterogeneous dN/dS across sites, model and parameters variable across genomic regions *(CoalEvol and INDELible)* |
|  | MG94 codon models (Muse and Gaut 1994), model and parameters constant within a genomic region *(CoalEvol)* |
|  | MG94 codon models, model and parameters variable across genomic regions *(CoalEvol)* |
|  | HB codon models (Halpern and Bruno 1998; Holder, Zwickl, and Dessimoz 2008), model and parameters constant within a genomic region *(CoalEvol)* |
|  | HB codon models, model and parameters variable across genomic regions *(CoalEvol)* |
|  | Empirical codon models^1^, model and parameters constant within a genomic region *(CoalEvol)* |
|  | Empirical codon models^1^, model and parameters variable across genomic regions *(CoalEvol)* |
|  | Variable dN/dS per branch (e.g., Dutheil et al. 2012), model and parameters constant within a genomic region *(CoalEvol)* |
|  | Variable dN/dS per branch, model and parameters variable across genomic regions *(CoalEvol)* |
|  | GY94, MG94 or HB × Nucleotide models (JC, …, GTR) (see, Pond and Muse 2005), parameters (Ti/Tv and rates of change) constant within a genomic region *(CoalEvol)* |
|  | GY94, MG94 or HB × Nucleotide models (JC, …, GTR), parameters (Ti/Tv and rates of change) variable across genomic regions *(CoalEvol)* |
|  | Heterogeneous rate across sites (+Gsites) *(CoalEvol and INDELible)* |
|  | Heterogeneous rate across genomic regions (+Gregions) *(CoalEvol)* |
|  | Heterogeneous rate across both sites and regions (+Gregions +variable Gsites) *(CoalEvol)* |
|  | Proportion of invariable sites within a genomic region (+I) *(CoalEvol and INDELible)* |
|  | Proportion of invariable sites variable across genomic regions (variable +I) *(CoalEvol and INDELible)* |
|  | Codon frequencies (1x64) *(INDELible)* |
|  | Codon frequencies (3x4) *(CoalEvol and INDELible)* |
|  | Codon frequencies constant within a genomic region *(CoalEvol and INDELible)* |
|  | Codon frequencies variable across genomic regions *(CoalEvol and INDELible)* |
|  | Variable codon frequencies across sites by categories (CAT), within a genomic region *(CoalEvol)* |
|  | Variable codon frequencies across sites by categories (CAT), and variable across genomic regions *(CoalEvol)* |
|  | Genetic code^2^, constant within a genomic region *(INDELible)* |
|  | Genetic code^2^, variable across genomic regions *(INDELible)* |
|  | Codon indels (for insertion and deletion events: rates and models (NB, LAV, POW continuous, POW discrete and user-specified)), model and parameters constant within a genomic region *(INDELible)* |
|  | Codon indels (for insertion and deletion events: rates and models (NB, LAV, POW continuous, POW discrete and user-specified)), model and parameters variable across genomic regions *(INDELible)* |
| Amino acid substitution | Empirical codon models^3^, model and parameters constant within a genomic region *(CoalEvol and INDELible)* |
|  | Empirical codon models^3^, model and parameters variable across genomic regions *(CoalEvol and INDELible)* |
|  | Amino acid frequencies constant within a genomic region *(CoalEvol and INDELible)* |
|  | Amino acid frequencies variable across genomic regions *(CoalEvol and INDELible)* |
|  | Variable Amino acid frequencies across sites by categories (CAT), within a genomic region *(CoalEvol)* |
|  | Variable Amino acid frequencies across sites by categories (CAT), and variable across genomic regions *(CoalEvol)* |
|  | Heterogeneous rate across sites (+Gsites) *(CoalEvol and INDELible)* |
|  | Heterogeneous rate across genomic regions (+Gregions) *(CoalEvol)* |
|  | Heterogeneous rate across both sites and regions (+Gregions +variable Gsites) *(CoalEvol)* |
|  | Proportion of invariable sites within a genomic region (+I) *(CoalEvol and INDELible)* |
|  | Proportion of invariable sites variable across genomic regions (variable +I) *(CoalEvol and INDELible)* |
|  | Amino acid indels (for insertion and deletion events: rates and models (NB, LAV, POW continuous, POW discrete and user-specified)), model and parameters constant within a genomic region *(INDELible)* |
|  | Amino acid indels (for insertion and deletion events: rates and models (NB, LAV, POW continuous, POW discrete and user-specified)), model and parameters variable across genomic regions *(INDELible)* |

^1^Empirical codon substitution models: ECMrest (Kosiol, Holmes, and Goldman 2007), ECMunrest (Kosiol, Holmes, and Goldman 2007) and ECMSchn2005 (Schneider, Cannarozzi, and Gonnet 2005) and any user-specified model.

^2^Genetic codes: Standard; Vertebrate Mitochondrial; Yeast Mitochondrial; Mold, Protozoan, and Coelenterate Mitochondrial/Mycoplasma/Spiroplasma; Invertebrate Mitochondrial; Ciliate, Dasycladacean and Hexamita Nuclear; Echinoderm and Flatworm Mitochondrial; Euplotid Nuclear; Bacterial and Plant Plastid; Alternative Yeast Nuclear; Ascidian Mitochondrial; Alternative Flatworm Mitochondrial; Blepharisma Nuclear; Chlorophycean Mitochondrial; Trematode Mitochondrial; Scenedesmus obliquus mitochondrial; Thraustochytrium Mitochondrial.

^3^Empirical amino acid substitution models: Blosum62 (Henikoff and Henikoff 1992), CpRev (Adachi et al. 2000), Dayhoff (Dayhoff, Schwartz, and Orcutt 1978), DayhoffDCMUT (Kosiol and Goldman 2005), HIVb (Nickle et al. 2007), HIVw (Nickle et al. 2007), JTT (Jones, Taylor, and Thornton 1992), JonesDCMUT (Kosiol and Goldman 2005), LG (Le and Gascuel 2008), Mtart (Abascal, Posada, and Zardoya 2007), Mtmam (Yang, Nielsen, and Masami 1998), Mtrev24 (Adachi and Hasegawa 1996), RtRev (Dimmic et al. 2002), VT (Muller and Vingron 2000), WAG (Whelan and Goldman 2001) and any user-specified model.

**Table S2.** Available statistical distributions in SGWE. Evolutionary parameters for sequence evolution (see Table S1) may change across regions according to these prior distributions.

| **Distribution** | **Description** | **Truncated** | **Examples** |
| --- | --- | --- | --- |
| Fix | Fixed value (integer or non-integer) | N.A.^1^ | fix 4; fix 0.7 |
| Uniform | Random between two values (integer or non-integer): lowest highest | N.A.^1^ | unif 1.0e-8 1.0e-5; unif 2e-9 5e-6; unif 0 3 |
| Normal | Normal distribution (mean, sd) | Yes | norm 1.0e-8 1.0e-5; norm 1.0e-8 1.0e-5 t 1.0e-9 1.0e-6 |
| Exponential | Exponential distribution (rate) | Yes | exp 1.0e-7; exp 1.0e-7 t 1.0e-8 1.0e-6 |
| Gamma | Gamma distribution (shape, rate “1/scale”) | Yes | gamma 1.0e-7 5.0e-7; gamma 1.0e-7 5.0e-7 t 2.0e-7 1.0e-6 |
| Beta | Beta distribution (shape1, shape 2) | Yes | beta 1.0e-7 5.0e-7; beta 1.0e-7 5.0e-7 t 2.5e-7 1.0e-6 |
| Dirichlet | Dirichlet distribution (alpha “vector”) | Yes | dirichlet 1 1 1 1; dirichlet 1 1 1 1 1 1 |

^1^The distribution cannot be truncated.

**Note S1. Validation of SGWE**

**i. Validation of the coalescent simulations**

The performance of SGWE’s coalescent simulation engine (*CoalEvol*) was compared with the theoretical expectations for the mean and variances of different simulation statistics, like the expected number of recombination events or time to the most recent common ancestor (TMRCA) (Table S3). We also checked whether these summary statistics agreed with those obtained with the *ms* program (Hudson 2002) under different evolutionary scenarios (see Table S3). *CoalEvol* simulates a number of intracodon recombination event that agrees with the theoretical expectations (Arenas and Posada 2010). In addition, simulated recombination breakpoints were accurately predicted by different analytical methods (Westesson and Holmes 2009; Marttinen et al. 2012).

**Table S3.** Simulated and expected number of recombination events and TMRCA for different levels of recombination. 200 replicates were simulated for each scenario. The symbol ± indicates standard error.

|  | SGWE | | Expected values | ms (Hudson’s simulator) | |
| --- | --- | --- | --- | --- | --- |
| *Simulated ρ* | *Mean of rec events (count.)* | *Mean TMRCA* | *Mean rec events (count.)* | *Mean rec events (all)* | *Mean TMRCA* |
| 0.0 | 0.0 | 0.9 ±0.0 | 0.0 | 0.0 ±0.0 | 0.9 ±0.0 |
| 0.5 | 1.4 | 1.0 ±0.0 | 1.4 | 1.0 ±0.1 | 1.0 ±0.0 |
| 5.0 | 14.0 | 1.5 ±0.1 | 14.1 | 14.0 ±0.5 | 1.5 ±0.1 |
| 10.0 | 28.9 | 1.9 ±0.1 | 28.3 | 31.0 ±0.8 | 1.8 ±0.1 |
| 50.0 | 141.0 | 2.7 ±0.1 | 141.4 | 248.0 ±3.8 | 2.8 ±0.1 |

In addition, simulations under user-specified population/species trees were manually checked by following the simulated evolutionary events. Longitudinal sampling was validated through simulations where samples were collected at different times and followed by a phylogenetic tree reconstruction.

**ii. Validation of the substitution models**

The program *jModelTest* (Posada 2008) correctly identified the following DNA substitution models simulated with SGWE (Table S4). This simulation consisted of 200 DNA alignments (25 sequences with length 999 nucleotides) with an average of nucleotide diversity (*Pi*) of approx 0.10. +I was simulated with a proportion of invariable sites = 0.5 and +G with a rate of heterogeneity (alpha shape) = 0.7. Models were selected using the Bayesian Information Criterion (BIC).

The program *ProtTest* (Abascal, Zardoya, and Posada 2005) always correctly identified the following amino acid substitution models simulated with SGWE (Table S5). Simulated alignments consisted in 8 sequences of 150 amino acids, under an amino acid substitution rate of 9.1x10^-4^. Models were selected using the Bayesian Information Criterion (BIC).

**Table S4.** Nucleotide substitution model prediction. The value for each model indicates the proportion of times the generating model was correctly identified.

| Substitution model | Selection of the true model (%) | Substitution model | Selection of the true model (%) |
| --- | --- | --- | --- |
| JC | 98.5 | HKY | 98.5 |
| JC+I | 85 | HKY+I | 77 |
| JC+G | 97 | HKY+G | 95.5 |
| K80 | 99.5 | SYM | 90.5 |
| K80+I | 80.5 | SYM+I | 66.5 |
| K80+G | 95.5 | SYM+G | 74 |
| F81 | 99 | GTR | 91.5 |
| F81+I | 81.5 | GTR+I | 69.5 |
| F81+G | 98.5 | GTR+G | 75.5 |

**Table S5.** Amino acid substitution model prediction. The value for each model indicates the proportion of times the generating model was correctly identified.

| Substitution model | Selection of the true model (%) | Substitution model | Selection of the true model (%) |
| --- | --- | --- | --- |
| Blosum62 | 100 | LG | 100 |
| CpRev | 100 | Mtart | 100 |
| Dayhoff | 100 | Mtrev | 100 |
| HIVb | 100 | Rtrev | 100 |
| HIVw | 100 | VT | 100 |
| JTT | 100 | WAG | 100 |

**iii. Estimation of the *dN*/*dS* ratio**

Different values of simulated nonsynonymous synonymous ratio rate (*dN/dS*) were accurately estimated with different methods included in the program *Hyphy* (Kosakovsky Pond, Frost, and Muse 2005) and *SNAP* (Korber 2000), (Table S6)*.*

**Table S6.** Simulated (*dN/dS*_sim_) and inferred ω values (using *Hyphy* and *SNAP*) for different diversity levels in the absence of recombination. ± indicate approximate 95% confidence intervals.

|  | *dN/dS*_sim_ = 0.20 | | *dN/dS*_sim_ = 1.00 | | ω*dN/dS*_im_ = 5.00 | |
| --- | --- | --- | --- | --- | --- | --- |
| *θ* | *SNAP* | *Hyphy* | *SNAP* | *Hyphy* | *SNAP* | *Hyphy* |
| 10 | 0.25 ±0.01 | 0.22 ±0.01 | 1.11 ±0.08 | 1.28 ±0.12 | 3.34 ±0.30 | 6.42 ±0.62 |
| 20 | 0.22 ±0.01 | 0.21 ±0.01 | 1.07 ±0.06 | 1.08 ±0.05 | 4.58 ±0.49 | 6.03 ±0.48 |
| 50 | 0.21 ±0.01 | 0.21 ±0.01 | 1.05 ±0.07 | 1.05 ±0.06 | 5.31 ±0.29 | 5.81 ±0.44 |
| 100 | 0.21 ±0.00 | 0.21 ±0.00 | 1.04 ±0.03 | 1.06 ±0.02 | 4.92 ±0.26 | 5.48 ±0.25 |
| 200 | 0.20 ±0.00 | 0.20 ±0.00 | 1.00 ±0.02 | 1.03 ±0.02 | 4.19 ±0.14 | 5.15 ±0.16 |

**Literature cited in the Supplementary Material**

Abascal F, Posada D, Zardoya R. 2007. MtArt: A New Model of Amino Acid Replacement for Arthropoda. *Mol Biol Evol*. 24:1-5.

Abascal F, Zardoya R, Posada D. 2005. ProtTest: selection of best-fit models of protein evolution. *Bioinformatics*. 21:2104-2105.

Adachi J, Hasegawa M. 1996. MOLPHY version 2.3: programs for molecular phylogenetics based in maximum likelihood. *Comput Sci Monogr*. 28:1-150.

Adachi J, Waddell PJ, Martin W, Hasegawa M. 2000. Plastid genome phylogeny and a model of amino acid substitution for proteins encoded by chloroplast DNA. *J Mol Evol*. 50:348-358.

Anisimova M, Bielawski JP, Yang Z. 2001. Accuracy and Power of the Likelihood Ratio Test in Detecting Adaptive Molecular Evolution. *Mol Biol Evol*. 18:1585-1592.

Arenas M, Posada D. 2010. Coalescent simulation of intracodon recombination. *Genetics*. 184:429-437.

Dayhoff MO, Schwartz RM, Orcutt BC. 1978. A model of evolutionary change in proteins. Pp. 345-352 *in* Dayhoff MO, ed. Atlas of protein sequence and structure, Washington D. C.

Dimmic MW, Rest JS, Mindell DP, Goldstein RA. 2002. rtREV: an amino acid substitution matrix for inference of retrovirus and reverse transcriptase phylogeny. *J Mol Evol*. 55:65-73.

Dutheil JY, Galtier N, Romiguier J, Douzery EJ, Ranwez V, Boussau B. 2012. Efficient selection of branch-specific models of sequence evolution. *Mol Biol Evol*. 29:1861-1874.

Fletcher W, Yang Z. 2009. INDELible: a flexible simulator of biological sequence evolution. *Mol Biol Evol*. 26:1879-1888.

Goldman N, Yang Z. 1994. A codon-based model of nucleotide substitution for protein-coding DNA sequences. *Mol Biol Evol*. 11:725-736.

Halpern AL, Bruno WJ. 1998. Evolutionary distances for protein-coding sequences: modeling site-specific residue frequencies. *Mol Biol Evol*. 15:910-917.

Henikoff S, Henikoff JG. 1992. Amino acid substitution matrices from protein blocks. *Proc Natl Acad Sci U S A*. 89:10915-10919.

Holder MT, Zwickl DJ, Dessimoz C. 2008. Evaluating the robustness of phylogenetic methods to among-site variability in substitution processes. *Philos Trans R Soc Lond B Biol Sci*. 363:4013-4021.

Hudson RR. 1998. Island models and the coalescent process. *Mol Ecol*. 7:413-418.

Hudson RR. 2002. Generating samples under a Wright-Fisher neutral model of genetic variation. *Bioinformatics*. 18:337-338.

Jones DT, Taylor WR, Thornton JM. 1992. The rapid generation of mutation data matrices from protein sequences. *Comput Appl Biosci*. 8:275-282.

Jukes TH, Cantor CR. 1969. Evolution of protein molecules. Pp. 21-132 *in* Munro HM, ed. Mammalian Protein Metabolism. Academic Press, New York, NY.

Kimura M, Weiss GH. 1964. The Stepping Stone Model of Population Structure and the Decrease of Genetic Correlation with Distance. *Genetics*. 49:561-576.

Korber B. 2000. HIV Signature and Sequence Variation Analysis. Pp. 55-72 *in* Rodrigo AG, Learn GH, eds. Computational Analysis of HIV Molecular Sequences. Kluwer Academic Publishers, Dordrecht, Netherlands.

Kosakovsky Pond SL, Frost SD, Muse SV. 2005. HYPHY: Hypothesis testing using phylogenies. *Bioinformatics*. 21:676-679.

Kosiol C, Goldman N. 2005. Different versions of the Dayhoff rate matrix. *Mol Biol Evol*. 22:193-199.

Kosiol C, Holmes I, Goldman N. 2007. An empirical codon model for protein sequence evolution. *Mol Biol Evol*. 24:1464-1479.

Lartillot N, Philippe H. 2004. A Bayesian mixture model for across-site heterogeneities in the amino-acid replacement process. *Mol Biol Evol*. 21:1095-1109.

Le SQ, Gascuel O. 2008. An improved general amino acid replacement matrix. *Mol Biol Evol*. 25:1307-1320.

Marttinen P, Hanage WP, Croucher NJ, Connor TR, Harris SR, Bentley SD, Corander J. 2012. Detection of recombination events in bacterial genomes from large population samples. *Nucleic Acids Res*. 40:e6.

Muller T, Vingron M. 2000. Modeling amino acid replacement. *J Comput Biol*. 7:761-776.

Muse SV, Gaut BS. 1994. A likelihood approach for comparing synonymous and nonsynonymous nucleotide substitution rates, with application to the chloroplast genome. *Mol Biol Evol*. 11:715-724.

Navascues M, Depaulis F, Emerson BC. 2010. Combining contemporary and ancient DNA in population genetic and phylogeographical studies. *Mol Ecol Resour*. 10:760-772.

Nickle DC, Heath L, Jensen MA, Gilbert PB, Mullins JI, Kosakovsky Pond SL. 2007. HIV-specific probabilistic models of protein evolution. *PLoS One*. 2:e503.

Pond SK, Muse SV. 2005. Site-to-Site Variation of Synonymous Substitution Rates. *Mol Biol Evol*. 22:2375-2385.

Posada D. 2008. jModelTest: phylogenetic model averaging. *Mol Biol Evol*. 25:1253-1256.

Schneider A, Cannarozzi GM, Gonnet GH. 2005. Empirical codon substitution matrix. *BMC Bioinformatics*. 6:134.

Tavaré S. 1986. Some probabilistic and statistical problems in the analysis of DNA sequences. Pp. 57-86 *in* Miura RM, ed. Some mathematical questions in biology - DNA sequence analysis. Amer Math Soc, Providence, RI.

Westesson O, Holmes I. 2009. Accurate detection of recombinant breakpoints in whole-genome alignments. *PLoS Comput Biol*. 5:e1000318.

Whelan S, Goldman N. 2001. A general empirical model of protein evolution derived from multiple protein families using a maximum-likelihood approach. *Mol Biol Evol*. 18:691-699.

Wiuf C, Posada D. 2003. A coalescent model of recombination hotspots. *Genetics*. 164:407-417.

Wright S. 1931. Evolution in Mendelian populations. *Genetics*. 16:97-159.

Yang Z, Nielsen R, Goldman N, Pedersen A-MK. 2000. Codon-substitution models for heterogeneous selection pressure at amino acid sites. *Genetics*. 155:431-449.

Yang Z, Nielsen R, Masami H. 1998. Models of amino acid substitution and applications to mitochondrial protein evolution. *Mol Biol Evol*. 15:1600-1611.
